# Metabolic Shock in Elderly Pertrochanteric or Intertrochanteric Surgery. Comparison of Three Surgical Methods. Is there a Much Safer?

**DOI:** 10.2478/rjaic-2020-0016

**Published:** 2020-12-31

**Authors:** Gómez-Garrido David, Bisaccia Michele, Ripani Umberto, Florin Cioancă, Schiavone Andrea, Ciotta Alfonso, Ibañéz -Vicente Cristina, Medina-Lorca Maria, Herrera-Molepecers Juan Antonio, Rollo Giuseppe, Meccariello Luigi

**Affiliations:** 1 Orthopedics and Traumatology Unit, Quironsalud Toledo Hospital, Toledo, Spain; 2 Orthopedics and Traumatology Unit, Department of Surgical and Biomedical Science, S.M. Misericordia Hospital, University of Perugia, Sant'Andrea delle Fratte, Perugia, Italy; 3 PainTherapy Center, Division of Anesthesia, Analgesia and Intensive Care, Department of Emergency, Ospedali Riuniti di Ancona, Ancona, Italy; 4 Department of Orthopedics and Traumatology, Emergency Clinical County Hospital of Bistrita, Bistrita, Romania; 5 Department of Advanced Medical and Surgical Sciences - University of Campania Luigi Vanvitelli Naples, Italy; 6 Department of General Medicine, "Hospital de Getafe", Madrid, Spain; 7 Hospital 12 de Octubre, Madrid, Spain; 8 Division of Orthopedics and Trauma Surgery, "Complejo Hospitalario Universitario de Toledo", Toledo, Spain; 9 Department of Orthopedics and Traumatology, Vito Fazzi Hospital, Lecce, Italy

**Keywords:** Surgical Trauma, Metabolic Shock, Intramedullary Nailing, Gliding Hip Screw, Minimally Invasive Technique, Interleukins

## Abstract

**Introduction:**

Trochanteric fractures are a major trauma in the elderly population and represent a significant part of public health spending. Various fixation devices are used as treatment for these fractures. This study aimed to evaluate three surgical methods in the treatment of pertrochanteric femoral fractures.

**Materials and methods:**

From January 1, 2013, to December 31, 2014, 86 patients were divided into 3 groups. Fifteen patients were treated with osteosynthesis by reamed intramedullary nailing (RIMN), 15 patients were treated with unreamed intramedullary nailing (UIMN), and 13 patients were treated with dynamic hip screw (DHS) plate osteosynthesis. All patients were >75 years of age. They were evaluated with a clinical radiological follow-up and laboratory examination (LDH, CPK, IL-1-B, IL-8, TNF-α, alpha-1-acid glycoprotein, D-dimer, fibrinogen, ESR, CRP, and procalcitonin).

**Results:**

IL-8, TNF-α, fibrinogen, D-dimer and alpha-1-acid glycoprotein levels were higher in the DHS group compared with the other two groups at 1 month after surgery (P<0.05). LDH, IL-1β, and IL-6 levels were higher in the DHS group compared with the other two groups at 3 months after surgery (P<0.05). From 3 to 6 months after surgery, the TNF-α level was high in the DHS and RIMN groups (P<0.05). Infection markers did not demonstrate a difference among the 3 groups. Twelve patients died during the 12-month follow-up. Regardless of the method used, morbidity and mortality are linked to enticement and comorbidities rather than surgery within 48 hours after the trauma.

**Conclusions:**

From our study, we can affirm that the values of cytokines and interleukins observed remain high during the 12-month follow up, regardless of whatever fixation devices or surgery type was performed within 48 hours of injury. Inflammatory markers are higher in patients in the DHS group. This can probably be explained by the fact that DHS technique is performed by open surgery, and this can create a higher inflammation of soft tissue. Mortality is reduced in the first 30 days after surgery if patients are mobilized early. Therefore, mortality in our study population of patients aged >75 years is linked more to the chronic inflammatory state and comorbidities, rather than fixation device or surgical type used.

However, future studies are needed to answer further questions that go beyond the scope of our study.

## Introduction

Pertrochanteric or intertrochanteric fractures are increasing in the population with an incidence of 57.5% [1]. Therefore, they are a common cause of hospitalization, disability, and mortality. In fact, 29% of patients lose the capacity to independently perform activities of daily living and 56% lose walking ability [2].

In 2014, Barbour et al. reported that the highest levels of inflammatory markers are correlated with an increased risk of fracture in elderly people [3].

At present, there are various surgical techniques available to treat pertrochanteric or intertrochanteric hip fractures, such as reamed intramedullary nailing (RIMN), unreamed intramedullary nailing (UIMN), and dynamic hip screw (DHS) [4].

The objective of this article is to analyze the positive and negative outcomes of these techniques and to determine if metabolic shock is related to a specific technique.

## Materials and Method

From September 2016 to May 2018, 43 patients (Table 1) were enrolled in this study.

**Table 1:** Description of population

	RIMN	UINM	DHS
Numbers	15	15	13
Average age (years)	83.5	84.9	82.4
Range of age (years)	75-100	75-102	75-96
Sex	12 M; 18 F	11 M; 12 F	8 M; 18 F
Antiplatelet used for thromboembolic prophylaxis	Enoxaparin: 15	Enoxaparin: 15	Enoxaparin: 13
Average score of thromboembolic risk according to card TVP Tuscany Region	5,7	5,9	5,5
Anesthetic risk (ASA)	III	III	III
Number of patients on therapy for osteoporosis before trauma	21	22	19
Average time of surgery (min)	23.6	18.6	45.3
Length of surgical wound (cm)	9.3	9.2	12.2

These patients were divided into three groups according to surgical technique. Fifteen patients were treated with RIMN, 15 patients were treated with UIMN, and 13 patients were treated with DHS (Table 1). All treated patients were at least 75 years old; had fractures classified as A1, A2, or A3, according to the AO classification; and had undergone the operation within 48 hours of the trauma. Patients younger than 75 years and those who used corticosteroids, immunosuppressants, anticoagulants, and antiplatelets were excluded from the study.

The patients in our study exhibited several comorbidities, such as cardiovascular disease, respiratory disease, renal disease, stroke, diabetes mellitus, rheumatoid disease, Parkinson’s disease, severe mental deterioration, Paget’s disease, and current smokers. In all three groups, 50% patients had up to three comorbidities. Comorbidity should be added as a high anesthetic risk because of the nature of the comorbidities and older age of the patients (Table 2).

**Table 2:** Comorbidities in the three investigated groups.

Comorbidity	RIMN	UIMN	DHS
Cardiovascular disease	6	7	6
Stroke	4	3	2
Respiratory disease	6	8	5
Renal disease	8	6	5
Diabetes mellitus	4	5	3
Rheumatoid disease	2	1	2
Parkinson’s disease	1	2	1
Severe mental deterioration in old age	8	8	5
Paget’s disease	1	1	0
Current smokers	5	4	2
**No. of comorbidities**			
1	5 (16.67%)	4 (15.33%)	4 (15.38%)
2	10 (33.33%)	11 (30.67%)	8 (30.76%)
≥3	15 (50%)	15 (50%)	14 (53.86%)

The patients treated with intramedullary nailing were able to walk, on an average, 2.3 (range: 2–4) days after the surgery, whereas the patients treated by DHS walked, on an average, 17.3 (range: 14–20) days after the surgery.

All patients were informed in a clear and comprehensive manner about the three types of treatments and the corresponding surgical alternatives. Patients were treated according to the ethical standards of the Declaration of Helsinki and were invited to read, understand, and sign the informed consent form. All patients had a follow-up of 1 year with clinical examinations and radiographic control evaluated at 1, 3, 6, and 12 months. We analyzed the metabolic shock with LDH and CPK metabolic shock; the inflammation with IL-1B, IL-8, TNFα, and alpha-1-acid glycoprotein; the thrombosis with D-dimer and fibrinogen; and the risk of infection with ESR, CRP, and procalcitonin. We do not consider the UI of transfused blood because transfusion must be performed according to the specific needs of the patient, and not to standardized levels.

### Western blotting analysis

The extraction of total proteins was performed with cells at a concentration of about 2.5 × 10^5^ cells/mL. Briefly, the cells were centrifuged at 1,200 rpm for 5 min at 4°C in order to remove the culture medium, and the pellet was washed with 1 mL of PBS 1×. Then it was lysed with about 200 μL of the lysis buffer (50 mM Tris-HCl, 150 mM NaCl, 10 mM NaF, and NP-40 1%) in the presence of protease inhibitors cocktail (PIC; Sigma-Aldrich) and 200 μM of phenylmethylsulfonyl fluoride (PMSF; Sigma-Aldrich). At the end, the samples were centrifuged for 15 min at 13,000 rpm at 4°C. The antibodies used were TNF (Santa Cruz) and IL-6, IL-8, and IL-10 (Abcam); Erk (Santa Cruz) was used to normalize the results.

The protein quantification was performed following the method of Bredford. The Biorad protein assay was based on the change in the color of the dye Coomassie Brilliant Blue G-250 in response to various concentrations of protein. The reagent binds mainly to the aromatic amino acid residues or bases, such as arginine, as standard was used a solution of bovine serum albumin to a known concentration. The reading was carried out in a spectrophotometer at a wavelength of 595 nm as it develops a color tending toward blue, the intensity of which is directly proportional to the protein concentration present in the samples. Image J was used to quantify the expression of proteins.

### Statistical analysis

Descriptive statistics were used to summarize the characteristics of the study group and subgroups, including means and standard deviations of all continuous variables. The t test was used to compare continuous outcomes. The Fisher, in this groups are smaller than 10 patients, exact test were used to compare Categorical variables. The statistical significance was defined as p<0.05. Pearson correlation coefficient (r) was used to compare the predictive score of outcomes and quality of life. Statistical analyses were performed using SPSS v.15.0 (SPSS Inc., an IBM Company, Chicago, IL, USA). Mean ages (and their standard deviations) of the patients were rounded at the closest year. The predictive score of outcomes and quality of life and their standard deviations were approximated at the first decimal, whereas these are approximated at the second decimal for Pearson correlation coefficient (r).

## Results

Initial measurement of inflammatory markers in the emergency room revealed no statistically significant difference among the three groups. However, all parameters analyzed were higher than baseline at 12-month follow-up. The value of LDH was higher in the DHS group compared with the other two groups (Table 3). CPK did not show a significant difference among the three groups during the follow-up (Table 4). IL-1β and IL-6 (Tables 5 and 6) levels were higher in the DHS group at 3 months after surgery (P<0.05). IL-8 level was higher in the DHS group compared with the other two groups at 1 month after surgery (Table 7). In the DHS group, TNF-α level was higher compared with the other two groups at 1 month after surgery (Table 7). From 3 to 6 months after surgery (Table 8), the TNF-α level was high in the DHS and RIMN groups (P<0.05). There were slightly higher values for alpha-1-acid glycoprotein in the DHS group at 1 month after surgery (Table 9). Fibrinogen and D-dimer levels were higher in the DHS group at 1 month after surgery (P<0.05) (Tables 10 and 11). Infection markers did not demonstrate a difference among the three groups (Tables 12–14). Because of the short-term therapy with antibiotics, fixation only caused an increase in inflammatory markers postoperatively. Twelve patients died during the 12-month follow-up.

**Table 3:** Trend of LDH changes (n.v. 0-600 U/L) during 1 year of follow-up in the three groups.

Time	Average LDH (U/L) RIMN	Average LDH (U/L) UIMN	Average LDH (U/L) DHS
0	787.3	786.2	788.1
Before surgery	370.5	376.3	368.8
After surgery	701.2	702.3	929.5
1st month	326.5	327.3	338.9
3rd month	250.6	284.4	256.7
6th month	232.5	237.9	236.4
12th month	372.6	377.1	382.3

**Table 4:** Trend of CPK changes (n.v 60-190 U/L) during 1 year of follow-up in the three groups.

Time	Average CPK (U/L) RIMN	Average CPK (U/L) UIMN	Average DHS CPK (U/L)
0	308.7	315.5	299.8
Before surgery	270.3	271.8	268.3
After surgery	388.3	368.2	408.2
1st month	202.3	198.3	199.4
3rd month	199.7	197.3	198.2
6th month	163.3	168.3	172.4
12th month	102.3	106.4	105.3

**Table 5:** Trend of IL-1β changes (n.v.<1pg/mL) during 1 year of follow-up in the three groups.

Time	Average IL-1β (pg/mL) RIMN	Average IL-1β (pg/mL) UINM	Average IL-1β (pg/mL) DHS
0	2.1	2.3	2.1
Before surgery	2.3	2.6	2.4
After surgery	3.8	3.9	4.4
1st month	2.7	2.7	3.1
3rd month	1.8	1.9	2.9
6th month	1.6	1.4	1.5
12th month	1.7	1.7	1.6

**Table 6:** Trend of IL-6 changes (n.v.<1.4pg/mL) during 1 year of follow-up in the three groups.

Time	Average IL-6 (pg/mL) RIMN	AverageIL-6 (pg/mL) UINM	AverageIL-6 (pg/mL) DHS
0	4.5	4.6	4.5
Before surgery	3.8	4.2	4.4
After surgery	6.5	5.6	6.7
1st month	2.4	2.9	4.4
3rd month	2.3	2.2	4.2
6th month	1.9	1.6	1.8
12th month	1.8	1.6	1.8

**Table 7:** Trend of IL-8 changes (n.v.<11 pg/mL) during 1 year of follow-up in the three groups.

Time	Average IL-8 (pg/mL) RIMN	Average IL-8 (pg/mL) UINM	Average IL-8 (pg/mL) DHS
0	18.3	17.6	16.9
Before surgery	24.5	23.3	22.5
After surgery	38.3	28.3	46.4
1st month	33.4	24.5	28.8
3^rd^ month	24.5	23.3	27.6
6th month	16.6	14.8	17.8
12th month	13.2	13.3	14.2

**Table 8:** Trend of alpha-1-acid glycoprotein changes (n.v. 55-120 mg/dL) during 1 year of follow-up in the three groups.

Time	Average alpha-1-acid glycoprotein (mg/dL) RIMN	Average alpha-1-acid glycoprotein (mg/dL) UINM	Average alpha-1-acid glycoprotein (mg/dL) DHS
0	237.5	245.6	248.2
Before surgery	280.2	288.5	274.6
After surgery	296.3	289.1	303.4
1st month	184.4	168.7	209.1
3rd month	156.8	148.9	178.2
6th month	193.8	198.1	199.2
12th month	181.4	182.6	185.7

**Table 9:** Trend of fibrinogen changes (n.v. 200-400 mg/dL) during 1 year of follow-up in the three groups.

Time	Average fibrinogen (mg/dL) RIMN	Average fibrinogen (mg/dL) UINM	Average fibrinogen (mg/dL) DHS
0	670.3	668.3	667.4
Before surgery	653.9	651.3	652.8
After surgery	710.3	711.3	708.9
1st month	458.3	459.4	600.5
3rd month	234.2	233.4	236.4
6th month	202.4	202.5	207.4
12th month	223.6	237.4	238.5

**Table 10:** Trend of D-dimer changes (n.v. 50-500 ng/mL) during 1 year of follow-up in the three groups.

Time	Average D-dimer (ng/mL) RIMN	Average D-dimer (ng/mL) UINM	Average D-dimer (ng/mL)DHS
0	724.3	726.9	727.5
Before surgery	798.3	795.4	789.6
After surgery	803.5	804.7	808.3
1st month	526.3	528.4	630.3
3rd month	182.4	180.3	184.2
6th month	78.3	79.5	80.5
12th month	77.3	78.8	79.5

**Table 11:** Trend of ESR changes (n.v. fino a 10 mm/h) during 1 year of follow-up in the three groups.

Time	Average ESR (mm/h) RIMN	Average ESR (mm/h) UINM	Average ESR (mm/h) DHS
0	54.2	56.3	55.2
Before surgery	64.3	68.4	64.9
After surgery	78.3	78.5	77.2
1st month	20.4	19.5	21.8
3rd month	16.3	17.8	20.3
6th month	12.3	11.8	12.2
12th month	11.9	12.3	11.4

**Table 12:** Trend of CRP changes (n.v.<5 mg/dL) during 1 year of follow-up in the three groups.

Time	Average CRP (mg/dL) RIMN	Average CRP (mg/dL) UINM	Average CRP (mg/dL) DHS
0	25.8	26.3	23.3
Before surgery	45.8	45.4	43.2
After surgery	59.5	59.3	51.8
1st month	12.1	12.8	12.2
3rd month	8.5	8.3	7.6
6th month	6.1	6.3	5.9
12th month	5.6	5.4	5.8

**Table 13:** Trend of procalcitonin changes (n.v. <0.05 ng/ML) during 1 year of follow-up in the three groups.

Time	Average procalcitonin (ng/mL) RIMN	Average procalcitonin (ng/mL) UINM	Average procalcitonin (ng/mL)DHS
0	0.01	0.01	0.01
Before surgery	0.03	0.04	0.05
After surgery	0.50	0.50	0.50
1st month	0.02	0.01	0.02
3rd month	0.01	0.01	0.01
6th month	0.02	0.01	0.01
12th month	0.01	0.01	0.02

**Table 14:** Trend of mortality during 1 year of follow-up in the three groups.

Time	Mortality RIMN	Mortality UINM	Mortality DHS
0	0	0	0
Before surgery	0	0	0
After surgery	0	0	1
1st month	2	1	4
3rd month	3	3	2
6th month	1	2	2
12th month	2	1	0
Total	8 (26.67%)	7 (23.33)	9 (34.61)

## Discussion

There are many different implants and operative procedures available to treat pertrochanteric or intertrochanteric fractures; however, choosing the best treatment is a topic of debate. Current data show an increasing use of cephalomedullary nails despite evidence in the literature suggesting the dynamic hip screw to be superior [5]. Intertrochanteric hip fractures in the elderly are frequent [6]. Unstable fracture pattern (fracture 31-A2 and 31-A3, AO/ASIF classification) occurs more frequently with increased age and low bone mineral density [6]. Unstable fracture can be difficult to manage, particularly in noncompliant patients with implant failure [6]. We used the common and widely used implant by Orthopedics and Traumatology within our study [6-7]. There is considerable debate about the optimal timing of hip fracture repair and whether delaying the repair affects outcomes. Postoperative rates of medical complications and death are high for many reasons. Current guidelines [8] recommend that surgeons perform hip fracture surgery within 48 hours of injury. Observational studies suggest earlier surgery is associated with better functional outcome and lower rates of nonunion, shorter hospital stays and duration of pain, and lower rates of complications and mortality. In published series, patients who underwent surgery earlier had lower rates of nonunion, avascular necrosis of the femoral head, urinary tract infections, decubitus ulcers, pneumonia, venous thromboembolism, and death and better long-term functional status than those who underwent surgery later [8].

In addition, delaying surgery prolongs patient’s pain and suffering. In a recent prospective cohort study involving 1,206 patients, those who underwent surgery within 48 hours had significantly fewer days of severe and very severe pain and shorter lengths of hospital stay. Higher pain ratings in patients with hip fracture are associated with longer postoperative lengths of stay, delayed postoperative rehabilitation, and increased risk of delirium, which increases the mortality and complications in elderly hospitalized patients [9]. If a fractured femur in elderly patients is considered as a polytrauma or with comorbidity, it receives an average score of 15–17 points on the Injury Severity Score scale, which may lead to multiple organ failure (MOF), acute respiratory distress syndrome (ARDS), and other serious complications [10]. In addition, the window of opportunity in patients with multiple trauma or multiple comorbidities to avoid a second HIT caused by the surgery is between 4 and 7 days from the trauma [11]. In fact, the mortality was 9.6% at 30 days after the surgery and 33% at 1 year from the surgery. Patients aged >75 years who need surgery to repair a hip fracture have a critically higher incidence of congestive heart failure [12]. In order to minimize complications or mortality in surgery, a kind of damage control can be carried out using gastric protectors, using antithrombotic therapy, and maintaining a good hemodynamic equilibrium with hemoglobin values >8 mg/dL [12]. However, 1-month mortality is reduced if patients are operated within 48 hours after the trauma [13]. Unfortunately, after 80 years of age, the surgery for hip fracture is carried out more so for the purpose of pain reduction, rather than to alter a patient’s perspective of life.

Hezman et al. [14] analyzed 9,157 intramedullary nail procedures and 27,687 plate fixation procedures from 1998 to 2007. During this period, the proportion of intertrochanteric hip fractures treated with intramedullary nail fixation, instead of plate fixation, increased from 3.3% to 63.1% [14]. Patients treated with an intramedullary nail had a higher adjusted risk of pulmonary embolism at 3 months after surgery (39%) and mortality at 1 year after surgery (9%) compared with those treated with plate fixation [14]. However, patients treated with an intramedullary nail had a 22% lower adjusted risk of conversion to hip replacement at 1 year after surgery. On the basis of the subgroup analysis of 4074 patients treated with an intramedullary nail and 2869 patients treated with plate fixation from 2006 to 2007, the lower adjusted risk of conversion to hip replacement at 1 year after surgery was still observed with intramedullary nails (~36%). The previously mentioned higher risk of pulmonary embolism and mortality associated with treatment with an intramedullary nail in the larger study group was not found in the subgroup analysis. Of the selected complications, deep venous thrombosis (4%) and mortality (25%) were the most frequently reported complications at 90 days and 1 year after surgery [14]. In our study, mortality is higher in the DHS group compared with the other groups (Table 3). However, at the end of the year of follow-up, there was not a statistically significant difference among the three groups, but the DHS group demonstrated slightly higher mortality compared with the other two groups. On the basis of our results, we believe that mortality is linked with the comorbidity of the patients instead of the surgical procedure. Introducing early weight bearing activity may assist in decreasing the mortality rate. According to Carretta et al. [15], patients with a hip fracture should have surgery within 2 days from admission in order to reduce 30-day mortality. Hip fractures have long been associated with significant morbidity and mortality, regardless of the surgical mode of treatment, with 1-year mortality rates reported to range from 13% to 32.5% [16, 17, 18, 19, 20, 21, 22]. In elderly patients, the pertrochanteric fracture might have an important impact on the entire body, which may present like a polytrauma for some patients [23]. For these patients, mainly elderly with multiple comorbidities, surgery represents the ‘‘second hit’’ after the first fracture trauma. Minimally invasive methods for indirect reduction and fixation [24] try to minimize the impact of this second hit. Unfortunately, to date, the expected benefit (decreased additional tissue damage) of these minimally invasive techniques [25] has not been objectively measurable. Elderly patients who have a pertrochanteric or intertrochanteric fracture and associated comorbidity may exhibit an injury severity score >15 and, therefore, are at risk for developing MOF or ARDS as in patients with polytrauma [26]. In our data, the values of risk of infection, such as ESR and CRP, were higher in all three groups during the perioperative period but had nonspecific markers. Procalcitonin, which is a more specific marker of sepsis and infection, was high in all three groups (P>0.05) in the perioperative period during the antibiotic prophylaxis, as reported in the literature [27]. A meta-analysis of polytrauma has shown that the increased risk of thrombosis is primarily due to medical conditions, such as a high ISS, blood transfusions, neurological deficit, hip injuries, and head trauma [28]. Many patients in our groups had this comorbidity. In a meta-analysis of the 27,441 patients with hip fracture, 449 (1.6%) developed deep venous thrombosis (DVT) or pulmonary embolism (PE). There was a significant difference in the rates of DVT/ PE based on surgery type (P = 0.015). Patients undergoing intramedullary nailing of inter-/peri-/subtrochanteric femoral fractures had the highest rates of DVT/PE (2.06%). However, the multivariate analysis revealed that renal failure and recent surgery were significant risk factors for DVT/PE [29]. In opposition to the previous literature, we demonstrate that for the first 3 months after surgery, the risk of DVT/PE was greater in the DHS group (P<0.05) compared with the other groups. However, the values remained high throughout the follow-up. Perhaps, this result is due to the prolonged recovery of patients in the DHS group compared with the other two groups. Many authors argued that the shock is due to a chaotic imbalance between the demand and the supply of energy substrate at cellular level [26]. In fact, the LDH was higher in the DHS group the day after the surgery. This may be due to the invasive nature of the surgery or patients’ comorbidities. CPK values were higher than normal because of the presence of sarcopenia syndrome in elderly patients [30]. There are several interrelationships between bone and muscle, and when the aging process affects one of these, the functionality of the other is compromised [30]. In experimental studies, it has been shown that the cytokines IL-6, IL-1β, and TNF-α exert effects on skeletal homeostasis [31]. In particular, IL-1β stimulates the proliferation of osteoblasts and production of mineralized bone matrix and inhibits the proliferation and differentiation of chondrocytes. It has also been reported that levels of IL-1β are higher in stabilized, rather than non-stabilized, fractures of the tibia in mice [31]. According to our data, the values of IL-6 and IL-1β are higher in the DHS group compared with the other two groups until the sixth month after surgery because the failure to engage in early weight bearing is correlated with the delay in bone healing [32]. TNF-α is a cytokine involved in systemic response and a member of a group of cytokines that stimulate the acute-phase reaction. It is mainly produced by macrophages, although it can be produced by CD4+ T lymphocytes, NK cells, neutrophils, mast cells, eosinophils, and neurons. It promotes bone healing and increases protein breakdown in muscle and fat breakdown in the adipose tissue. High postoperative grades at 6 months follow-up in this group are due to the insult of RIMN on reaming marrow when compared with group’s values with UIMN [33]. We are not certain as to the explanation behind the trend observed for the values of the DHS group. The secretion of IL-8 is increased by the oxidative stress, which in turn causes the recruitment of inflammatory cells and a further increase in mediators of oxidative stress, making it a key parameter in localized inflammation. IL-8 is also known to be a potent promoter of angiogenesis [34]. IL-8 seems not to be useful for our purpose of quantifying surgical tissue [31] but may be useful to understand the oxidative stress of the body and quantify its ability to repair bone via angiogenesis. Alpha-1-acid glycoprotein is a marker too nonspecific to be taken into account. In fact, it turns out to also be present in inflammatory and microtraumatic states, such as Legg-Calve-Perthes disease [35]. Alpha-1-acid glycoprotein has been identified as one of the four potentially useful circulating biomarkers for estimating the 5-year risk of all-cause mortality (the other three are albumin, very low-density lipoprotein particle size, and citrate) [36]. Given the values in Table 15, perhaps, we can allude to why a higher mortality was observed in the DHS group compared with the other two groups, without the presence of any statistical difference among groups.

## Conclusions

From our study, we can affirm that the values of cytokines and interleukins observed remain high during the 12-month follow-up, regardless of whatever fixation devices or surgery type was performed within 48 hours of injury. Mortality is reduced in the first 30 days after surgery if the patients are mobilized early. Therefore, mortality in our study population of patients aged >75 years is linked more to the chronic inflammatory state and comorbidities, rather than fixation device or surgical type used.

However, future studies are needed to answer further questions that go beyond the scope of our study.

### Limitations in Investigational Methodology

The limitations of the current study were the limited number of patients, nonprobability sample of convenience because of few centric sample, and level 1 trauma center.

Another limit is that a part of population is from a retrospective study and another part is from a continuative case series.

Selection of patients may be biased, making generalization of results difficult. It may be unclear whether the confluence of findings is merely a chance occurrence or is truly characteristic of a new disease or syndrome. Case series studies have no comparison group and can only be used to generate hypotheses about the relationship between an exposure and an outcome.

Another limitation was that the measurements and intervention were made without randomization of the researcher to the experimental groups, which have the potential for bias. Finally, other limiting factors of the study acknowledged by the authors can be the potential for regression to the mean, the presence of temporal confounders, and the mention of subjective score.
